# Peripheral and autonomic nervous system involvement in spinocerebellar ataxia type 3: unveiling an invisible burden

**DOI:** 10.1007/s00415-025-13588-x

**Published:** 2026-01-07

**Authors:** Kristofoor E. Leeuwenberg, Nens van Alfen, Bart P. van de Warrenburg, Roderick P. P. W. M. Maas

**Affiliations:** Department of Neurology, Donders Institute for Brain, Cognition, and Behaviour, Radboud University Medical Center, Geert Grooteplein Zuid 10, 6525 GA Nijmegen, The Netherlands

**Keywords:** Spinocerebellar ataxia type 3, Peripheral nervous system, Autonomic dysfunction, Patient-reported outcomes, Disease burden

## Abstract

**Background:**

Neuropathological examinations in spinocerebellar ataxia type 3 (SCA3) have demonstrated peripheral and autonomic nervous system degeneration, but the impact of associated symptoms on genetically affected individuals at different disease stages remains understudied.

**Objective:**

To investigate the clinical burden of peripheral and autonomic nervous system involvement in SCA3 mutation carriers across the disease spectrum.

**Methods:**

Forty SCA3 mutation carriers, including ten pre-ataxic individuals, completed questionnaires about muscle cramps, neuropathic pain, autonomic symptoms, activities of daily living, and quality of life, and underwent a standardized clinical examination of ataxia and neuropathy severity. Data were compared with 16 healthy controls.

**Results:**

All but one of the ataxic and 60% of pre-ataxic individuals experienced muscle cramps at least weekly. Neuropathic pain was reported by 20% of pre-ataxic and 16.7% of ataxic mutation carriers, while the average number of autonomic symptoms in both groups was 2 and 4.7, respectively. Neuropathy severity scores were significantly higher in pre-ataxic and ataxic individuals than in healthy controls and associated with (i) worse self-reported functional status and (ii) clinician-reported ataxia severity. The number of autonomic symptoms was associated with patient-reported impairments in daily life and quality of life.

**Conclusion:**

Clinical features of peripheral and autonomic nervous system degeneration are very common in SCA3, may already be observed in pre-ataxic individuals, and independently contribute to patient-reported disease burden and clinician-rated overall ataxia severity.

**Supplementary Information:**

The online version contains supplementary material available at 10.1007/s00415-025-13588-x.

## Introduction

Spinocerebellar ataxia type 3 (SCA3) is a dominantly inherited neurodegenerative disorder caused by a CAG trinucleotide repeat expansion in the *ATXN3* gene. It represents one of the most common types of SCA worldwide and is characterized by an invariably progressive decline in functional capacity and quality of life [1–5]. Although cerebellar ataxia is the hallmark symptom of SCA3, a variety of extracerebellar and non-motor features contribute to its overall disease burden [6]. Among these are peripheral nervous system (PNS) involvement and autonomic dysfunction [7, 8].

Neuropathological studies in SCA3 have commonly shown involvement of PNS components, including the dorsal root ganglia, peripheral nerves, and anterior horn cells [9, 10]. Degeneration of these structures may not only induce unpleasant physical sensations, such as muscle cramps and neuropathic pain, but also contributes to sensory ataxia severity and therefore accelerates overall ataxia progression. Nonetheless, the impact of PNS involvement on daily functioning of SCA3 mutation carriers at different disease stages has received scant attention to date. Likewise, symptoms of autonomic dysregulation are not rare in SCA3, but commonly underrecognized in clinical practice [11]. A recent systematic review concluded that previous research on autonomic dysfunction in SCAs has been highly fragmented, with individual studies mostly focusing on distinct deficits [8]. As such, the overall impact of autonomic symptoms on daily life and their disease course remain largely unknown.

A detailed characterization of the symptomatic burden of PNS and autonomic nervous system (ANS) involvement is warranted in SCA3, also in light of ongoing therapeutic developments in polyglutamine ataxias. Although antisense oligonucleotides have been shown to reach PNS tissues, including the dorsal root ganglia [12], following intrathecal administration in animals, it is unclear whether these findings automatically translate into reliable, consistent, and effective penetration and targeting of these structures in humans. The primary objectives of the present study were to (1) determine the prevalence of muscle cramps, neuropathic pain, and symptoms of autonomic dysfunction in ataxic and pre-ataxic SCA3 mutation carriers and (2) evaluate the relationship between the severity of PNS and ANS involvement with overall ataxia severity, activities of daily living, and quality of life.

## Methods

### Study design and participants

We report the clinical baseline data from a prospective, observational single-center cohort study. Individuals were eligible for participation if they were 18 years or older, had a pathogenic repeat expansion in the *ATXN3* gene, and did not have a medical history of other conditions associated with neuropathy or myopathy. Based on their Scale for the Assessment and Rating of Ataxia (SARA) score, participants were classified as pre-ataxic (SARA < 3) or ataxic mutation carriers (SARA ≥ 3) [13]. Data from SCA3 participants were compared with healthy controls without a history of neurological or psychiatric disorders. The study was approved by the medical research ethics committee (MREC) Oost-Nederland. Written informed consent was obtained from all participants.

### Clinical assessment

The Muscle Cramp Scale (MCS) [14], Cramp Disability Scale (CDS) [15], Neuropathic Pain Scale (NPS) [16], and Total Neuropathy Score clinical version (TNSc) [17], including a total of 16 autonomic symptoms across cardiovascular, vasomotor, sudomotor, secretomotor, pupillomotor, gastrointestinal, and urinary domains, were used as outcome measures. In order to exclude other non-specific types of pain, the NPS was administered only if a screening question confirmed the presence of neuropathic pain characteristics (i.e., burning, painful cold, and/or electric shock-like sensations in the upper and/or lower limbs). All participants underwent a clinical examination by the same neurologist (R.M.), including the SARA and clinician-rated section of the TNSc.

### Statistical analysis

Data are reported as mean and standard deviation or frequency and percentage, as appropriate. Differences between healthy controls and SCA3 mutation carriers were analyzed using Fisher’s exact tests for categorical data (effect size: odds ratio [*OR*]) and Mann–Whitney *U* tests for continuous variables (effect size: rank-biserial correlation [*rrb*]). Spearman correlation coefficients (ρ) were used to examine associations between the aforementioned outcome measures and predicted time to/from disease onset, SARA score, EQ-5D-5L visual analogue scale (VAS) score, Friedreich Ataxia Rating Scale – Activities of Daily Living (FARS-ADL) subscore, and Patient-Reported Outcome Measure of Ataxia (PROM-Ataxia) score [18–20]. Predicted time to/from disease onset was determined by subtracting the estimated age of onset, derived from the expanded allele’s repeat length [21], from the subject’s current age. Multivariable linear regression analyses were subsequently performed to evaluate if PNS and ANS indices independently affect ataxia severity, activities of daily living, and quality of life, adjusting for disease duration and repeat length. Age significantly increased variance inflation factors and was therefore excluded from the models. Statistical analyses were performed using R studio (Version 2024.04). The level of significance was set at *p* < 0.05.

## Results

Data were collected from 40 SCA3 mutation carriers, including 30 ataxic and 10 pre-ataxic individuals, and 16 healthy controls. Demographic and clinical characteristics of participants are outlined in Table 1. Compared to healthy controls (1.4 ± 1.5), higher TNSc scores—indicating more severe neuropathy—were not only found in the ataxic group (13.3 ± 4.6; *p* < 0.001, *rrb* = 0.97) but also already in pre-ataxic SCA3 mutation carriers (5.7 ± 2.7; *p* < 0.001, *rrb* = 0.80).

**Table 1:** Demographic and clinical characteristics of the participants in this study

	SCA3 mutation carriers			Healthy controls
	Pre-ataxic	Ataxic	Combined	
Number of participants	10	30	40	16
Age (years)	42.4 ± 7.2	58.1 ± 7.5	54.2 ± 10.1	56.5 ± 9.1
Male/female	5/5	15/15	20/20	9/7
CAG repeat length	66.5 ± 3.0	66.6 ± 2.8	66.6 ± 2.8	–
Predicted disease duration (years)^a^	−1.2 ± 6.8	14.7 ± 7.1	10.7 ± 9.8	–
SARA score	1.8 ± 0.5	13.5 ± 5.4	10.6 ± 6.9	0.5 ± 0.7
TNSc score	5.7 ± 2.7	13.3 ± 4.6	11.4 ± 5.4	1.4 ± 1.5

*SCA3* spinocerebellar ataxia type 3, *SARA* Scale for the Assessment and Rating of Ataxia, *TNSc* Total Neuropathy Score clinical version

^a^According to the equation by Tezenas du Montcel and colleagues based on CAG repeat length and actual age [21]

### Prevalence of muscle cramps, neuropathic pain, and autonomic symptoms in SCA3

**Fig. 1:**
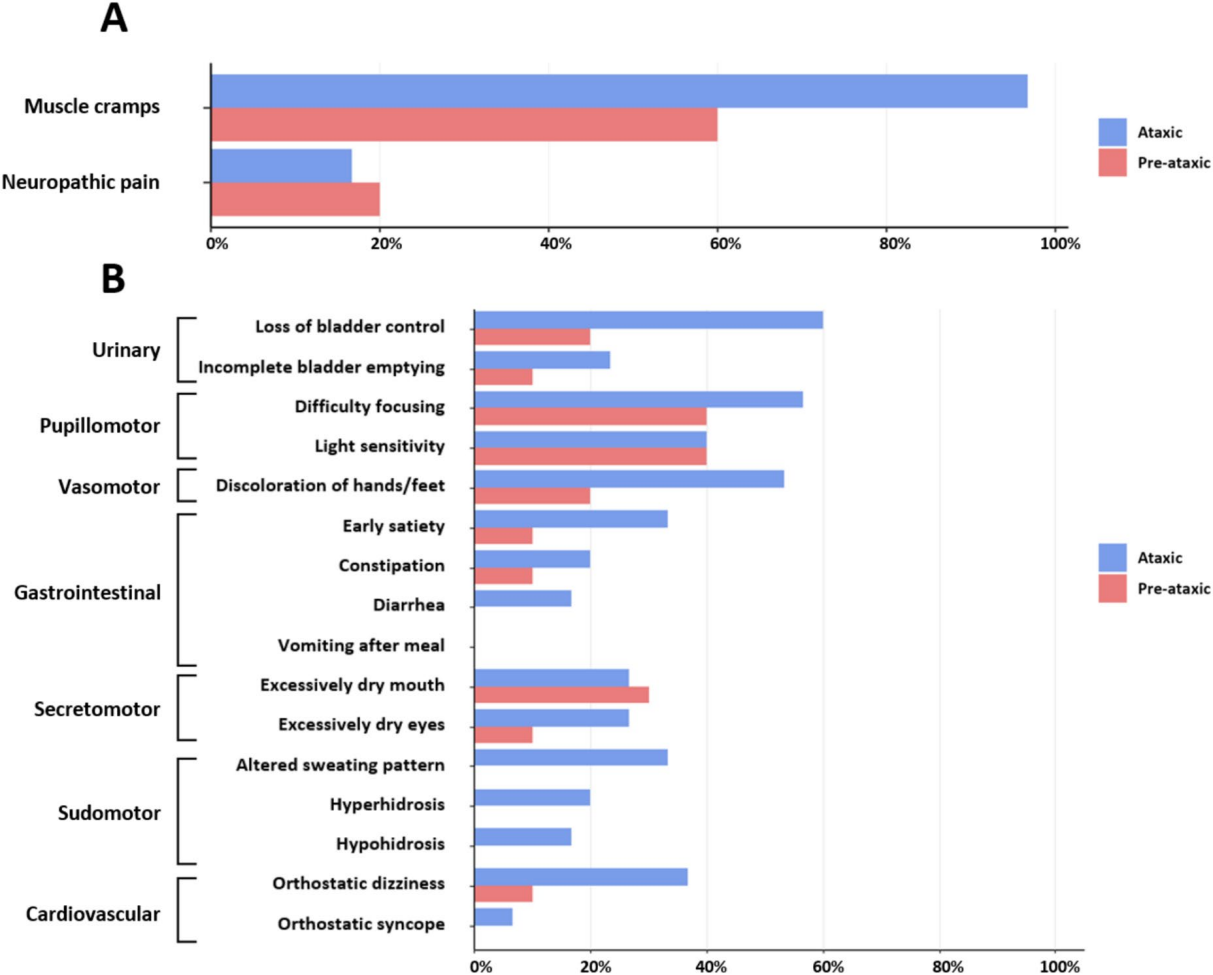
Prevalence of muscle cramps, neuropathic pain, and symptoms of autonomic dysfunction in pre-ataxic and ataxic SCA3 mutation carriers

The MCS revealed that 60% of pre-ataxic and 96.7% of ataxic participants experienced muscle cramps at least weekly (Fig. 1A), most commonly in the legs (80%), followed by the arms (32.5%), neck (15%), and trunk (12.5%). The overall prevalence of cramps was significantly higher in both the ataxic (*p* < 0.001, *OR* = 100.57) and pre-ataxic (*p* = 0.046, *OR* = 5.97) subgroups compared with healthy controls. Moreover, 25% of SCA3 mutation carriers reported moderate to severe impairments in daily life due to muscle cramps, as indicated by disruptions of work or sleep. Neuropathic pain was reported by 20% of pre-ataxic and 16.7% of ataxic individuals (mean severity score 38/100).

Autonomic involvement was common in SCA3, with an average number of 4.7 and 2 symptoms in ataxic and pre-ataxic mutation carriers, respectively. Urinary (67.5%) and pupillomotor (65%) domains were most commonly affected, followed by vasomotor (45%), gastrointestinal (42.5%), secretomotor (40%), sudomotor (37.5%), and cardiovascular domains (32.5%) (Fig. 1B).

Compared with healthy controls, the MCS score and number of autonomic symptoms were significantly higher in both the ataxic subgroup (*p* < 0.001, *rrb* = 0.94 and 0.93) and the pre-ataxic subgroup (*p* = 0.033 and < 0.001, respectively, *rrb* = 0.54 and 0.76). A detailed overview of results is provided in Supplementary Tables 1–3.

### Associations with activities of daily living, quality of life, and ataxia severity

**Fig. 2:**
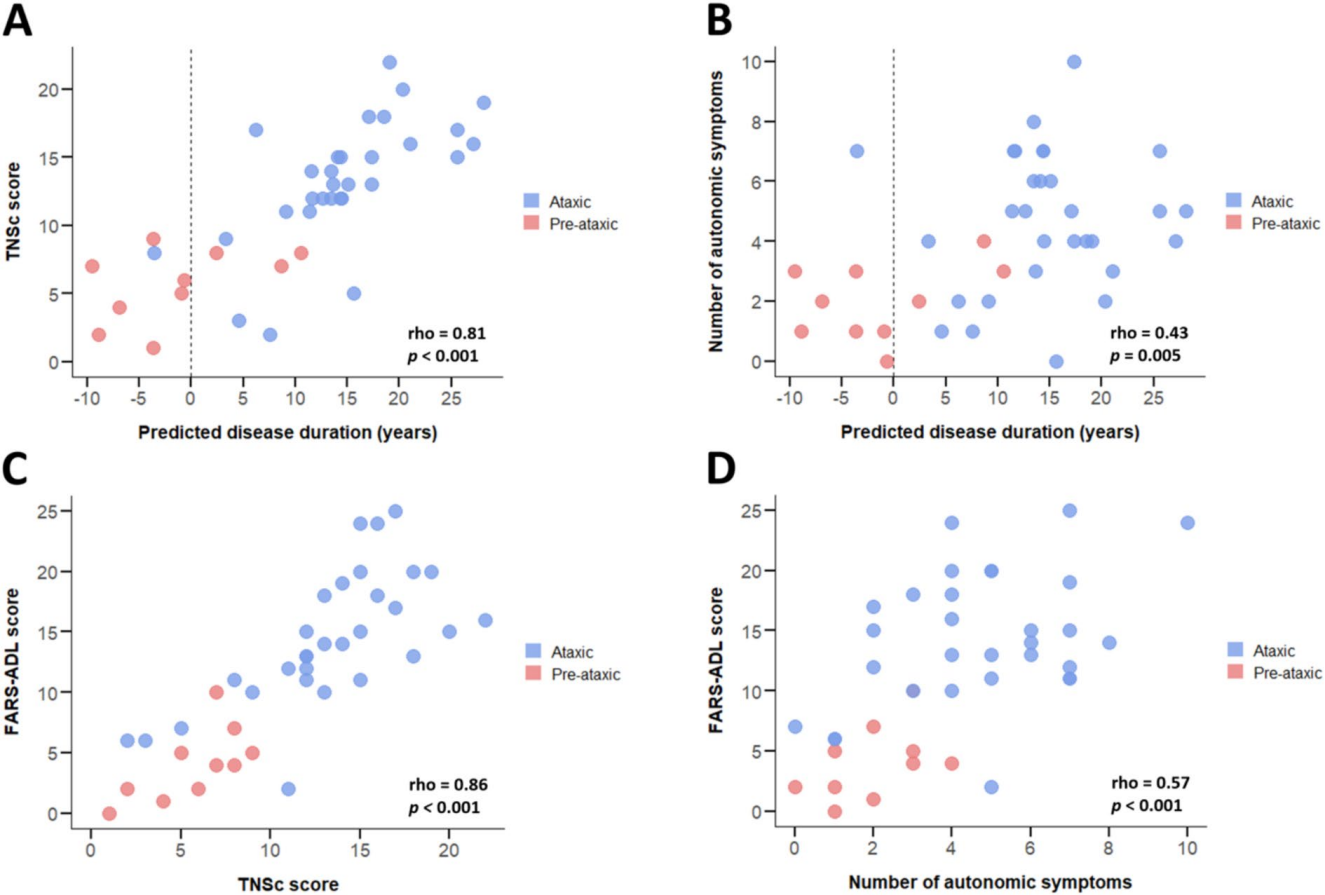

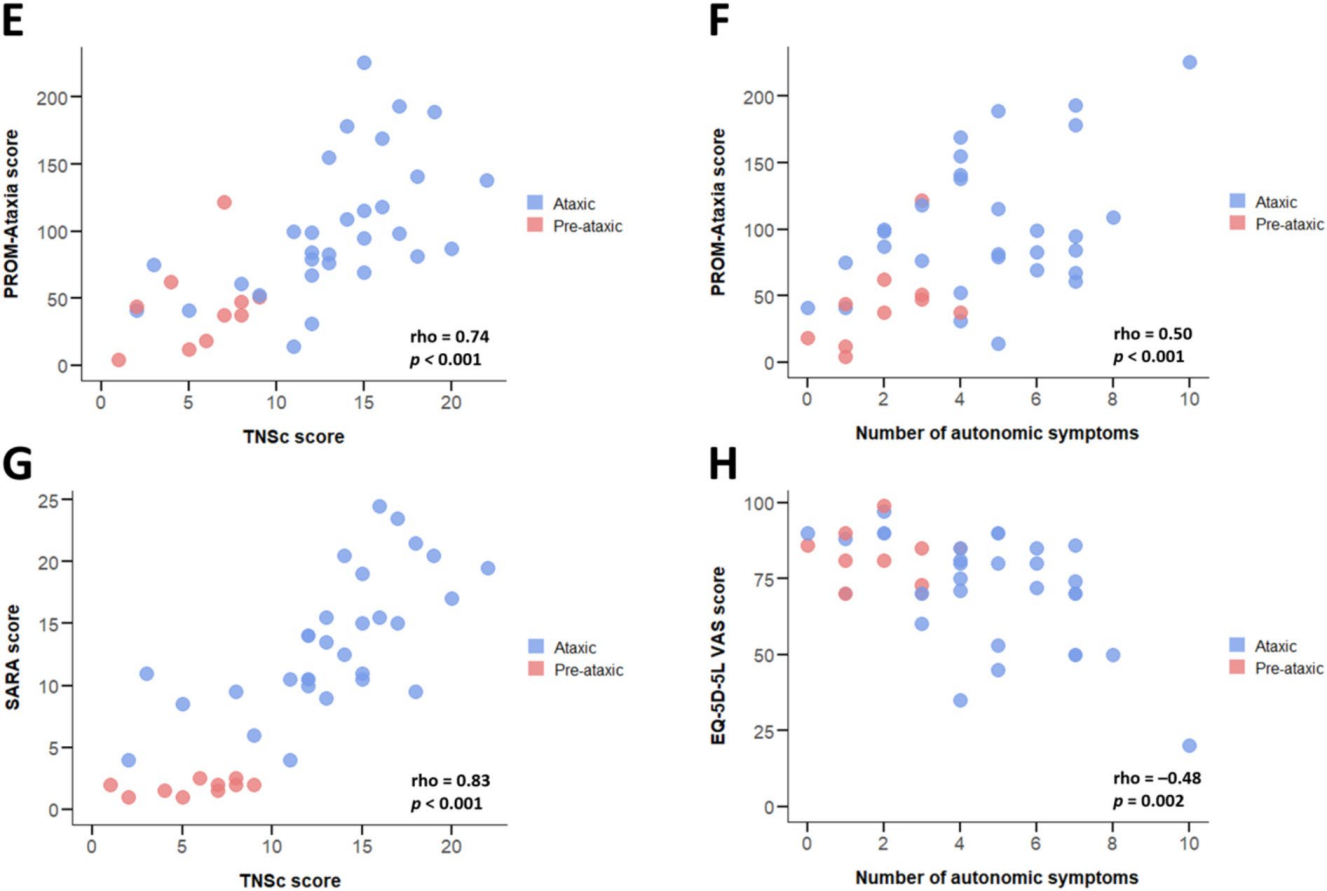
Impact of peripheral nervous system involvement and autonomic symptoms in pre-ataxic and ataxic SCA3 mutation carriers. Shown are associations with predicted disease duration (**A** and **B**), self-reported activities of daily living (**C** and **D**), self-reported overall functioning and wellbeing (**E** and **F**), ataxia severity (**G**), and health-related quality of life (**H**). *TNSc* Total Neuropathy Score clinical version, *FARS*-*ADL* Friedreich Ataxia Rating Scale-Activities of Daily Living, *PROM*-*Ataxia* Patient-Reported Outcome Measure of Ataxia, *SARA* Scale for the Assessment and Rating of Ataxia, *VAS* visual analogue scale

TNSc scores strongly correlated with predicted time to/from onset (ρ = 0.81, *p* < 0.001), functional impairment (FARS-ADL: ρ = 0.86, PROM-Ataxia: ρ = 0.74, both *p* values < 0.001), and ataxia severity (SARA: ρ = 0.83, *p* < 0.001) (Fig. 2). The number of autonomic symptoms showed a moderate correlation with predicted time to/from onset (ρ = 0.43, *p* = 0.005), functional impairment (FARS-ADL: ρ = 0.57, PROM-Ataxia: ρ = 0.50, both *p* values < 0.001), and quality of life (EQ-5D-5L VAS: ρ =  −0.48, *p* = 0.002). No significant correlation was observed between MCS scores and predicted time to/from onset.

After controlling for disease duration and repeat length, multivariable linear regression analyses showed that TNSc score was independently associated with SARA score (*B* = 0.72; *SE* = 0.20; *p* = 0.001; adjusted *R*^2^ = 0.72). Model outcomes imply that each one-unit increase in TNSc score is paralleled by an average 0.72 point increase in SARA score. Using similar adjustments, TNSc scores were also independently related to FARS-ADL (*B* = 0.72; *SE* = 0.21; *p* = 0.002; adjusted *R*^2^ = 0.68) and PROM-Ataxia scores (*B* = 7.14; *SE* = 2.18; *p* = 0.002; adjusted *R*^2^ = 0.46). Finally, the number of autonomic symptoms was independently associated with EQ-5D-5L VAS scores (*B* = −3.83; *SE* = 1.12; *p* = 0.002; adjusted *R*^2^ = 0.46).

## Discussion

Integrating patient-reported and clinician-reported measures, this study aimed to examine the impact of peripheral and autonomic nervous system involvement on SCA3 mutation carriers across the disease spectrum. Our findings indicate that symptoms and signs of PNS and ANS degeneration are very common in SCA3, start early, and make an important contribution to overall disease burden. Both the severity of neuropathy and the number of autonomic symptoms are associated with greater impairments in daily life and, in the case of autonomic symptoms, also with quality of life. These symptoms may already start during the pre-ataxic stage and tend to increase gradually with disease progression.

Our data confirm that muscle cramps are highly prevalent in SCA3, affecting 87.5% of mutation carriers (and even 96.7% of ataxic individuals) in our cohort at least weekly versus 80–82% in previous studies [15, 22]. Cramps were not only reported in the lower limbs, but also commonly involved upper limb, neck, and trunk muscles. Interestingly, muscle cramps occurred significantly more often in pre-ataxic SCA3 mutation carriers than in healthy controls who were, on average, almost 15 years older, suggesting early PNS involvement. The underlying mechanism is believed to involve collateral nerve sprouting following lower motor neuron loss with subsequent changes in motor axonal excitability properties [15, 22]. The early occurrence of cramps, combined with only gradual progression over time, may account for the rather weak associations that we observed with other disease outcomes. From a clinical perspective, the appearance of muscle cramps already during early disease stages should prompt physicians to periodically evaluate these bothersome symptoms, starting from the first outpatient encounter, and inform patients about the available non-pharmacological and pharmacological treatment options [23].

Although less common than muscle cramps, our data reveal that neuropathic pain is present in one-sixth of SCA3 mutation carriers, also including pre-ataxic individuals. Because of the study’s focus on PNS-related symptoms, we specifically evaluated the presence and severity of neuropathic pain (after a targeted screening question) and did not include other types of pain that might be more prevalent [2, 24, 25]. This highlights the clinical importance of detailed pain characterization in SCA3 mutation carriers to guide appropriate treatment.

An important finding of our study is the association between a more severe neuropathy and higher number of autonomic symptoms on the one hand and a worse self-reported functional status on the other hand. Even after accounting for disease duration and repeat length as potential confounders, the TNSc score and number of autonomic symptoms remained significantly associated with patient-reported outcomes, highlighting their impact on daily life. Additionally, a higher autonomic symptom count was linked to reduced patient-reported quality of life, which emphasizes the need for regular screening during outpatient clinic visits.

In addition to an association with relevant patient-reported outcome measures, this study provides empirical evidence for a relationship between clinical neuropathy severity and SARA score in SCA3, irrespective of disease duration and repeat length. Our findings thus not only corroborate an important sensory ataxia component in SCA3—alongside the established cerebellar degeneration—but also seem to underscore that involvement of peripheral nerves and dorsal root ganglia independently contributes to overall ataxia severity.

Our study has several limitations. Particularly for autonomic symptoms, an additional objective assessment—beyond patient report—would have been valuable. Furthermore, a comprehensive investigation of other types of pain would have enhanced the understanding of this frequently debilitating symptom in SCA3 and could have allowed comparisons with previous studies. Finally, the sample size of our single-center study limits detailed subgroup analyses, particularly in pre-ataxic individuals.

In conclusion, PNS-related symptoms and features suggesting autonomic dysregulation constitute a significant part of the (invisible) disease burden of SCA3 mutation carriers, already during early disease stages, and should not be overlooked in clinical and scientific practice. Moreover, concomitant signs of PNS involvement in this disease importantly contribute to overall ataxia severity, as evaluated with SARA. Because SCA3 is a multisystem disorder, disease monitoring and future therapeutic developments should encompass the full extent of its manifestations.

## Supplementary Information

Below is the link to the electronic supplementary material.

Supplementary file1 (DOCX 17 KB)

Supplementary file2 (DOCX 16 KB)

Supplementary file3 (DOCX 19 KB)

## Data Availability

Anonymized data will be shared by the corresponding author upon reasonable request from a qualified investigator.
