# Supplementary material for: Peripheral and autonomic nervous system involvement in spinocerebellar ataxia type 3: unveiling an invisible burden
Source: J Neurol. 2026 Jan 7;273(1):64. doi: 10.1007/s00415-025-13588-x (PMC12779735; doi:10.1007/s00415-025-13588-x)
Supplement: Supplementary file 1 — Supplementary file1 (DOCX 17 KB) [file 415_2025_13588_MOESM1_ESM.docx]

**Supplementary Table 1.** Muscle cramps in SCA3 mutation carriers and healthy controls.

|  | | **SCA3 mutation carriers** | | | **Healthy controls** N = 16 |
| --- | --- | --- | --- | --- | --- |
|  |  | *Pre-ataxic* N = 10 | *Ataxic* N = 30 | *All* N = 40 |  |
| **Muscle Cramp Scale (MCS)** | | | | |  |
| Triggering of muscle cramps | (1) None with any activity, *n* (%) (2) Rarely with strenuous activity, *n* (%) (3) Frequently with strenuous activity, *n* (%) (4) Almost always with strenuous activity, *n* (%) (5) Almost always with any activity, *n* (%) | 2 (20%) 5 (50%) 2 (20%) 0 (0%) 1 (10%) | 3 (10%) 13 (43.3%) 9 (30%) 2 (6.7%) 3 (10%) | 5 (12.5%) 18 (45%) 11 (27.5%) 2 (5%) 4 (10%) | 8 (50%) 8 (50%) 0 (0%) 0 (0%) 0 (0%) |
| Frequency of muscle cramps | (1) None a week, *n* (%) (2) Less than 3 in a week, *n* (%) (3) On average once a day, *n* (%) (4) Less than 5 a day, *n* (%) (5) More than 5 a day, *n* (%) - Comparison with healthy controls (% with value ≥ 2) | 4 (40%) 4 (40%) 0 (0%) 2 (20%) 0 (0%) *p* = 0.046 OR = 5.97 | 1 (3.3%) 20 (66.7%) 5 (16.7%) 3 (10%) 1 (3.3%) *p* < 0.001 OR = 100.57 | 5 (12.5%) 24 (60%) 5 (12.5%) 5 (12.5%) 1 (2.5%) *p* < 0.001 OR = 27.44 | 13 (81.3%) 3 (18.7%) 0 (0%) 0 (0%) 0 (0%) - |
| Location of cramps | (1) Right arm, *n* (%) (2) Left arm, *n* (%) (3) Right leg, *n* (%) (4) Left leg, *n* (%) (5) Trunk, *n* (%) (6) Neck, *n* (%) | 2 (20%) 0 (0%) 7 (70%) 5 (50%) 1 (10%) 0 (0%) | 11 (36.7%) 11 (36.7%) 25 (83.3%) 22 (73.3%) 4 (13.3%) 6 (20%) | 13 (32.5%) 11 (27.5%) 32 (80%) 27 (67.5%) 5 (12.5%) 6 (15%) | 1 (6.3%) 0 (0%) 7 (43.7%) 4 (25%) 0 (0%) 0 (0%) |
| Number of cramp areas | (1): One area, *n* (%) (2): Two areas, *n* (%) (3): Three areas, *n* (%) (4): Four areas, *n* (%) (5): Everywhere, *n* (%) | 7 (70%) 2 (20%) 1 (10%) 0 (0%) 0 (0%) | 7 (23.3%) 13 (43.3%) 3 (10%) 5 (16.7%) 2 (6.7%) | 14 (35%) 15 (37.5%) 4 (10%) 5 (12.5%) 2 (5%) | 16 (100%) 0 (0%) 0 (0%) 0 (0%) 0 (0%) |
| Severity of most cramps | (1) Unrecognized sensation, *n* (%) (2) Minor discomfort, *n* (%) (3) Massage can be used to stop cramps, *n* (%) (4) Massage cannot easily stop cramps, *n* (%) (5) Nearly continuous cramps, *n* (%) | 3 (30%) 3 (30%) 4 (40%) 0 (0%) 0 (0%) | 2 (6.7%) 18 (60%) 8 (26.7%) 1 (3.3%) 1 (3.3%) | 5 (12.5%) 21 (52.5%) 12 (30%) 1 (2.5%) 1 (2.5%) | 10 (62.5%) 4 (25%) 2 (12.5%) 0 (0%) 0 (0%) |
| Muscle cramps affecting overall daily activities | Numeric Rating Scale (0 – 10) | 0.4 ± 1.0 | 1.6 ± 2.1 | 1.3 ± 1.9 | 0 |
| MCS total score  - Comparison with healthy controls | | 8.2 ± 3.6 *p* = 0.033  *rrb =* 0.54 | 11.4 ± 3.9 *p* < 0.001 *rrb =* 0.94 | 10.6 ± 4.0 *p* < 0.001 *rrb* = 0.84 | 5.3 ± 1.3 - |
| **Cramp Disability Scale (CDS)** | | | | |  |
| To what extent do muscle cramps limit daily activities? | (1) No muscle cramps, *n* (%) (2) Mild, *n* (%) (3) Moderate, *n* (%) (4) Severe, *n* (%) | 4 (40%) 4 (40%) 2 (20%) 0 (0%) | 1 (3.3%) 21 (70%) 6 (20%) 2 (6.7%) | 5 (12.5%) 25 (62.5%) 8 (20%) 2 (5%) | 13 (81.3%) 3 (18.7%) 0% 0% |
| **Medication use for muscle cramps** | | | | | |
| 4 participants used magnesium supplements  1 participant used tizanidine 2 mg four times per day 1 participant used cannabis oil once per day | | | | | |

SCA3 = Spinocerebellar ataxia type 3; MCS = Muscle Cramp Scale; CDS = Cramp Disability Scale; *rrb* = rank-biserial correlation
