# Supplementary material for: Peripheral and autonomic nervous system involvement in spinocerebellar ataxia type 3: unveiling an invisible burden
Source: J Neurol. 2026 Jan 7;273(1):64. doi: 10.1007/s00415-025-13588-x (PMC12779735; doi:10.1007/s00415-025-13588-x)
Supplement: Supplementary file 2 — Supplementary file2 (DOCX 16 KB) [file 415_2025_13588_MOESM2_ESM.docx]

**Supplementary Table 2.** Neuropathic pain in SCA3 mutation carriers and healthy controls.

|  | | **SCA3 mutation carriers** | | | **Healthy controls** N = 16 |
| --- | --- | --- | --- | --- | --- |
|  |  | *Pre-ataxic* N = 10 | *Ataxic* N = 30 | *All* N = 40 |  |
| **Neuropathic Pain Scale (NPS)** | | | | | |
| Patients reporting neuropathic pain, *n* (%) | | 2 (20%) | 5 (16.7%) | 7 (17.5%) | 0 (0%) |
| Characteristics of reported pain  (Numeric Rating Scale 0 – 10) | (1) How intense is the pain? | 6.5 | 4.4 | 5.0 ± 1.7 | - |
|  | (2) How sharp is the pain? | 7.5 | 3.6 | 4.7 ± 3.0 | - |
|  | (3) How hot is the pain? | 2.5 | 3.8 | 3.4 ± 3.0 | - |
|  | (4) How dull is the pain? | 0 | 6.8 | 4.9 ± 4.3 | - |
|  | (5) How cold is the pain? | 0 | 3.0 | 2.1 ± 3.5 | - |
|  | (6) How sensitive is the skin to light touch? | 2.0 | 2.2 | 2.1 ± 2.2 | - |
|  | (7) How itchy is the pain? | 0 | 1.4 | 1.0 ± 2.6 | - |
|  | (9) How unpleasant is the pain? | 4.5 | 6.0 | 5.6 ± 2.0 | - |
|  | (10a) How intense is the deep pain? | 6.0 | 6.4 | 6.3 ± 1.9 | - |
|  | (10b) How intense is the surface pain? | 2.0 | 3.6 | 3.1 ± 2.7 | - |
| (8) Temporal pattern of reported pain | Occasional, *n* (%) Constant, *n* (%) | 1 (50%) 1 (50%) | 2 (40%) 3 (60%) | 3 (42.9%) 4 (57.1%) | - - |
| NPS score | Mean score of affected patients | 31 | 41 | 38 | - |
| **Daily use of pain medication** | | | | | |
| 1 participant used pregabalin 50 and 125 mg twice per day  1 participant used paracetamol 1000 mg twice per day Other participants took pain medication not daily, but as needed | | | | | |

SCA3 = Spinocerebellar ataxia type 3; NPS = Neuropathic Pain Scale.
