# Supplementary material for: Peripheral and autonomic nervous system involvement in spinocerebellar ataxia type 3: unveiling an invisible burden
Source: J Neurol. 2026 Jan 7;273(1):64. doi: 10.1007/s00415-025-13588-x (PMC12779735; doi:10.1007/s00415-025-13588-x)
Supplement: Supplementary file 3 — Supplementary file3 (DOCX 19 KB) [file 415_2025_13588_MOESM3_ESM.docx]

**Supplementary Table 3.** Autonomic symptoms in SCA3 mutation carriers and healthy controls.

|  | | **SCA3 mutation carriers** | | | **Healthy controls** N = 16 |
| --- | --- | --- | --- | --- | --- |
|  |  | *Pre-ataxic* N = 10 | *Ataxic* N = 30 | *All* N = 40 |  |
| **Presence of autonomic symptoms** | | | | | |
| Cardiovascular | Orthostatic dizziness, *n* (%) | 1 (10%) | 11 (36.7%) | 12 (30%) | 0 (0%) |
|  | Orthostatic syncope, *n* (%) | 0 (0%) | 2 (6.7%) | 2 (5%) | 0 (0%) |
|  | - At least 1 cardiovascular symptom, *n* (%) | 1 (10%) | 12 (40%) | 13 (32.5%) | 0 (0%) |
| Vasomotor | Color changes of the skin in hands/feet, *n* (%) | 2 (20%) | 16 (53.3%) | 18 (45%) | 1 (6.3%) |
| Sudomotor | Changes in sweating pattern, *n* (%) | 0 (0%) | 10 (33.3%) | 10 (25%) | 0 (0%) |
|  | Sweating significantly more, *n* (%) | 0 (0%) | 6 (20%) | 6 (15%) | 0 (0%) |
|  | Sweating significantly less, *n* (%) | 0 (0%) | 5 (16.7%) | 5 (12.5%) | 0 (0%) |
|  | - At least 1 sudomotor symptom, *n* (%) | 0 (0%) | 15 (50%) | 15 (37.5%) | 0 (0%) |
| Secretomotor | Dry eyes, *n* (%) | 1 (10%) | 8 (26.7%) | 9 (22.5%) | 0 (0%) |
|  | Dry mouth, *n* (%) | 3 (30%) | 8 (26.7%) | 11 (27.5%) | 0 (0%) |
|  | - At least 1 secretomotor symptom, *n* (%) | 3 (30%) | 13 (43.3%) | 16 (40%) | 0 (0%) |
| Pupillomotor | Sensitivity of the eyes for bright light, *n* (%) | 4 (40%) | 12 (40%) | 16 (40%) | 0 (0%) |
|  | Difficulty focusing of the eyes, *n* (%) | 4 (40%) | 17 (56.7%) | 21 (52.5%) | 1 (6.3%) |
|  | - At least 1 pupillomotor symptom, *n* (%) | 6 (60%) | 20 (66.7%) | 26 (65%) | 1 (6.3%) |
| Gastrointestinal | Early satiety after a meal, *n* (%) | 1 (10%) | 10 (33.3%) | 11 (27.5%) | 1 (6.3%) |
|  | Vomiting after a meal, *n* (%) | 0 (0%) | 0 (0%) | 0 (0%) | 0 (0%) |
|  | Episodes of diarrhea, *n* (%) | 0 (0%) | 5 (16.7%) | 5 (12.5%) | 0 (0%) |
|  | Episodes of constipation, *n* (%) | 1 (10%) | 6 (20%) | 7 (17.5%) | 2 (12.5%) |
|  | - At least 1 gastrointestinal symptom, *n* (%) | 2 (20%) | 15 (50%) | 17 (42.5%) | 2 (12.5%) |
| Urinary | Loss of bladder function, *n* (%) | 2 (20%) | 18 (60%) | 20 (50%) | 0 (0%) |
|  | Problems with emptying of the bladder, *n* (%) | 1 (10%) | 8 (23.3%) | 8 (20%) | 0 (0%) |
|  | - At least 1 urological symptom, *n* (%) | 3 (30%) | 20 (66.7%) | 23 (57.5%) | 0 (0%) |
| Number of autonomic symptoms per patient - Comparison with healthy controls | | 2.0 ± 1.2 *p* < 0.001 *rrb* = 0.76 | 4.7 ± 2.3 *p* < 0.001 *rrb* = 0.93 | 4.0 ± 2.4 *p* < 0.001 *rrb* = 0.89 | 0.3 ± 0.8 - |
| **Medication use for autonomic symptoms** | | | | | |
| 1 participant used solifenacin 5 mg once per day related to bladder loss control | | | | | |

SCA3 = Spinocerebellar ataxia type 3; *rrb* = rank-biserial correlation.
